# Military Dental Practice: Its Modifications and Limitations

**Published:** 1901-11

**Authors:** Henry D. Hatch

**Affiliations:** New York City


### MILITARY DENTAL PRACTICE: ITS MODIFICATIONS AND LIMITATIONS.[1]

BY HENRY D. HATCH, D.D.S., NEW YORK CITY.

Mr. President and Gentlemen,—What shall the new military practice consist of? What are its limitations and modifications? and How must the conservative civil practice be modified so as to best serve the interests of the army and the individual soldier? These are the questions with which it is the province of this paper to deal.

There are certain branches of dentistry, as it exists to-day, which

---

[1] Abstract of a paper read before the Section on Stomatology of the American Medical Association, at St. Paul, Minnesota, June, 1901.

it would seem wise to eliminate altogether,—namely, prosthetic dentistry, orthodontia, crown- and bridge-work, and gold filling.

Prosthetic dentistry would be impracticable for the following reasons:

First. The appliances necessary for the construction of artificial dentures are cumbersome and would add that much more to an already overburdened transport service.

Second. The time required to do such work is more than could be spared, owing to the few surgeons assigned to the service.

That orthodontia would have no place in military surgery is self-evident to any surgeon.

Crown- and bridge-work would hardly be feasible, except perhaps at certain army posts, for the reason that to be of any value it must be done with a certain nicety, requiring much time, many additional instruments, and much expensive material.

Gold filling is placed in this category for much the same reason. Gold filling, to be of any value, must be done under the most propitious circumstances, requiring a good chair, good assistants, dry, clean surroundings, many fine instruments, and plenty of time, the item of time being the most important in this case, as, with the present limited number of surgeons, to give the necessary time to one man would probably rob others of needed attention.

Eliminating, then, all the above, there still remains work enough for the most energetic and able men.

Let us now briefly glance at the positive side of the question. First, as to outfit. Other things being equal, the dental service will be popular in the army, as it takes up little room, demands few transportation facilities, and adapts itself to the prevailing conditions easily and uncomplainingly.

The outfit, then, should be as small as is consistent with good work, everything snugly packed and capable of being unpacked and packed again in the shortest possible time. One of the lessons taught by the Boers in their struggle for independence is that the modern army must return to the practice of Julius Cæsar, where luggage was reduced to the minimum. A portable head-rest attached to an ordinary chair would have to take the place of the regular dental chair, except at regular army posts. Anæsthetics could be given on the operating-table of the general surgeon, or on a cot-bed. The instrument-case could be mounted on a tripod or a table, and made to take the place of the usual cabinet.

An instrument sterilizer should, by all means, be made a part of the outfit, such as a Schering formaldehyde sterilizer, or the smaller one as modified by Dr. Stanton, of New York, or the formaldehyde sterilizer devised by Dr. Low, of Buffalo, which is perhaps more cheaply and easily operated.

Formaldehyde gas must prove to be the ideal sterilizing agent in military as well as in civil dental practice. It requires a very small quantity of spirits to generate the gas. As previously pointed out to the profession by the writer, boiling is the cheapest and most ready method of sterilizing where the nature of the instruments and appliances admits of boiling; but with dental instruments we have the mirrors, which cannot be boiled; also corundum wheels, engine hand-pieces, and many other things. But whatever the method, it is to be hoped that the dental surgeons will not lag behind the general surgeons in this respect.

OPERATIVE DENTISTRY.

The dental surgeon will at first be most often called upon to relieve pain, either by extracting or treating exposed pulps, alveolar abscesses, etc.; and here is where the high character of the men selected by our excellent examining board will have a chance to show itself, and prove to the army and the nation that the modern dental surgeon is something more than a mere tooth-puller.

Extracting may have to be done more often than in civil practice; but by using good judgment and adopting means adapted to the exigencies of the service, extracting may be reduced to the minimum.

Certain pulpless molar teeth, which, in private practice would be sterilized and filled to the ends of the roots, might be saved by using some of the mummifying methods, preferably Miller's, always filling over it with some filling that could easily be taken out, if need be, and the pulp-chamber and canals redressed. Or, in certain cases, extracting might be avoided by opening into the pulp-chamber at the cervix, and waiting until the case could have more thorough treatment. At any rate, root-filling should not be attempted unless the operator has the time and the facilities to do it thoroughly.

In extracting and other surgical work, it is to be hoped that anæsthetics, either local or general, will be used as they would be in civil practice. Even a soldier ought not to be called upon to endure more pain than is necessary. Besides, many will be either in the hospital or on the verge of it, when rough treatment and shock might be the last straw. Any tendency to become brutal or rough, on the part of the dental surgeon, should meet with prompt rebuke or dismissal from the service.

The dental surgeon will have plenty of opportunity to observe and treat diseased conditions of the soft and osseous tissues surrounding the teeth. If there are carefully observed and accurate records kept, much will be added to our meagre knowledge of these conditions. The dental surgeon will see conditions caused by both mercury and the need of mercury, and perhaps will be able to get truer histories in the army than out of it.

That dental surgery merges into oral and general surgery so that a dividing line can hardly be drawn, goes without saying. Hence, it is to be expected that the dental surgeon will have much to do with fractures and gun-shot wounds involving the maxillæ. The use of interdental splints made of vulcanite or swaged metal would hardly be applicable in military practice, for the reason given above concerning prosthetic work. In their place could be substituted something after the Angle system of easily adapted bands and ligatures, or an easily made splint consisting of metal bands connected by hard solder to heavy platinum and gold, or iridio-platinum, wire, the piece made loosely fitting on a cast gotten from a modelling composition impression and cemented in place. An interdental splint in many instances, taking the place of vulcanite, may be made quickly and easily by cooling and trimming two side "bites" of modelling composition. In cases of resection of a considerable portion of the inferior maxilla, such a side "bite" of modelling composition, put in place immediately, answers an excellent purpose.

### IDENTIFICATION BY THE TEETH.

For purposes of identification, after disaster by fire or flood, or other causes, where clothing is destroyed or the soft parts decomposed, experience has proved that identification by the teeth is the only reliable one. The condition of the bodies after the disaster to the battle-ship "Maine," and after the catastrophe of the Bazar de la Charité, in Paris, May 4, 1897, and many others, proves this. Therefore it is urged that the government be memorialized to the effect that all officers and men in the army and navy, and all who shall be mustered into the service, shall have charts and casts made of their teeth by the dental surgeons of the army, or others appointed for the purpose, such charts and casts to be properly inscribed and filed with the proper departments for reference in case of death or desertion.

It would seem that, with the training the dental surgeon has received in aseptic methods and in minor surgery, he might be an excellent assistant to the general surgeon in emergencies, after battles, etc., thereby tending to promote mutual regard, and a better understanding between the two professions, now separate, but destined to become one.

